# Crop plants as models for understanding plant adaptation and diversification

**DOI:** 10.3389/fpls.2013.00290

**Published:** 2013-08-01

**Authors:** Kenneth M. Olsen, Jonathan F. Wendel

**Affiliations:** ^1^Biology Department, Washington UniversitySt. Louis, MO, USA; ^2^Ecology, Evolution, and Organismal Biology Department, Iowa State UniversityAmes, IA, USA

**Keywords:** adaptation, artificial selection, association mapping, crop improvement, domestication syndrome, evolutionary genomics, parallel evolution

## Abstract

Since the time of Darwin, biologists have understood the promise of crop plants and their wild relatives for providing insight into the mechanisms of phenotypic evolution. The intense selection imposed by our ancestors during plant domestication and subsequent crop improvement has generated remarkable transformations of plant phenotypes. Unlike evolution in natural settings, descendent and antecedent conditions for crop plants are often both extant, providing opportunities for direct comparisons through crossing and other experimental approaches. Moreover, since domestication has repeatedly generated a suite of “domestication syndrome” traits that are shared among crops, opportunities exist for gaining insight into the genetic and developmental mechanisms that underlie parallel adaptive evolution. Advances in our understanding of the genetic architecture of domestication-related traits have emerged from combining powerful molecular technologies with advanced experimental designs, including nested association mapping, genome-wide association studies, population genetic screens for signatures of selection, and candidate gene approaches. These studies may be combined with high-throughput evaluations of the various “omics” involved in trait transformation, revealing a diversity of underlying causative mutations affecting phenotypes and their downstream propagation through biological networks. We summarize the state of our knowledge of the mutational spectrum that generates phenotypic novelty in domesticated plant species, and our current understanding of how domestication can reshape gene expression networks and emergent phenotypes. An exploration of traits that have been subject to similar selective pressures across crops (e.g., flowering time) suggests that a diversity of targeted genes and causative mutational changes can underlie parallel adaptation in the context of crop evolution.

## Introduction

The recognition that domesticated species serve as excellent models for studying morphological evolution can be traced to Charles Darwin, who famously opens his *Origin of Species* with a chapter devoted to “Variation under Domestication.” In his introduction, Darwin highlights the value of domesticated organisms for understanding the evolutionary process:

*At the commencement of my observations, it seemed to me probable that a careful study of domesticated animals and cultivated plants would offer the best chance of making out this obscure problem. Nor have I been disappointed; in this and in all other perplexing cases I have invariably found that our knowledge, imperfect though it be, of variation under domestication, offered the best and safest clue. I may venture to express my conviction of the high value of such studies, although they have been very commonly neglected by naturalists* (Darwin, 1859).

These comments turned out to be remarkably (but not surprisingly) prescient. The subsequent 150 years of advances in the fields of evolutionary and developmental biology have demonstrated that studies of domestication, particularly in plants, provide a wealth of insights into the genetic and developmental bases of morphological evolution. This is partly due to the central role that crops play in sustaining civilization. Crop species supply the vast majority of humankind's caloric intake, either directly as food or indirectly as livestock feed, and crop species have long been studied by breeders and other plant biologists for purposes of crop improvement. A result has been the development of a rich toolkit in many crops for studying the genetic basis of agronomically-related traits; these resources may include extensive germplasm collections, advanced generation pedigrees for use in genetic manipulations, and ever-expanding “omics” databases such as genome sequences and transcriptome, small RNA and proteome profiles in various tissues. A growing number crop species now feature annotated reference genome sequences [reviewed by Feuillet et al. (2011)], with at least ten reference genomes released since 2012 alone (e.g., D'Hont et al., 2012; Garcia-Mas et al., 2012; Mayer et al., 2012; Paterson et al., 2012; Sato et al., 2012; Varshney et al., 2012; Zhang et al., 2012a; Guo et al., 2013; Jia et al., 2013; Ling et al., 2013). Equally importantly, the recent time frame during which domestication has occurred (generally <10,000 years, with the origins of agriculture) means that for most crops, wild forms representing genetically close models of the actual ancestors still exist. The domesticated and progenitor forms can therefore be directly compared and crossed experimentally, providing insights into the molecular, developmental, and physiological impacts of selection during domestication. Together these features make many crops highly tractable model systems for studying genomic and phenotypic evolution during domestication.

An additional advantage of crop species for evolutionary analysis is the opportunity they provide for studying parallel evolution. The repeated evolution of adaptive traits is a hallmark of evolution; this phenomenon characterizes a wide spectrum of organisms from across the tree of life (Arendt and Reznick, 2008; Losos, 2011). Understanding the genetic and developmental mechanisms that underlie parallel adaptation has emerged as one of the key questions of modern evolutionary biology, as these analyses hold promise for revealing not only the mechanisms that underlie the origin of novel phenotypes, but also the nature of evolutionary constraints and the likelihood of specific evolutionary trajectories or processes. Crop species are eminently positioned for yielding insight into these questions, because selection during domestication has generated a suite of traits that are shared across many crop species (the “domestication syndrome”; Hammer, 1984; Harlan, 1992). These independently evolved traits can be studied at various levels of phylogenetic divergence, including separate lineages within a single crop species (e.g., fragrance in Asian rice; Kovach et al., 2009), different crop species within a single genus (e.g., grain color in Asian and African rice; Gross et al., 2010), and different genera at higher taxonomic levels (e.g., grain shattering in cereal crops; Paterson et al., 1995; Lin et al., 2012). Comparisons of independently domesticated crop lineages can thus facilitate inferences into the molecular and developmental underpinnings of parallel adaptation, providing insight into the relative roles of constraint and lability in shaping evolution.

As in many areas of biology, studies of crop domestication have undergone a quantum leap in the last decade with the development of massively parallel, next-generation sequencing (NGS) and related “omics” approaches. Advances have been particularly evident in research aimed at understanding the genotype–phenotype connection. The dense, genome-wide SNP marker coverage afforded by NGS genotyping [reviewed by Davey et al. (2011)] is now readily applied in genetic mapping of domestication-related traits, including in mapping populations derived from traditional biparental crosses (e.g., crop X wild parents), advanced intercrossed populations derived from diverse parental lines [e.g., nested association mapping (NAM); Buckler et al., 2009; McMullen et al., 2009; Larsson et al., 2013, and genome-wide association mapping in populations of unrelated individuals (GWAS); Ramsay et al., 2011; Harper et al., 2012; Huang et al., 2012b; Riedelsheimer et al., 2012]. Genome resequencing and/or genome-wide SNP scans are also being used to identify candidate genomic regions bearing molecular signatures of selection during domestication (e.g., low nucleotide diversity, augmented linkage disequilibrium) (He et al., 2011; Harper et al., 2012; Huang et al., 2012a; Cavanagh et al., 2013; Hufford et al., 2013). Unlike methods based on trait mapping, such selection screens do not require any a priori assumptions about the traits that were subject to selection during domestication, and they can thus potentially reveal genes underlying subtle phenotypic changes such as metabolic shifts (e.g., Hufford et al., 2012). Followed by fine mapping and functional characterization of candidate genes, these assorted mapping strategies are proving highly effective at revealing the molecular bases of domestication phenotypes.

As a complementary approach to genetic mapping, domestication-related changes in transcriptomes and gene expression networks can be explored to assess the genome-scale impacts of domestication on the emergent plant phenotype (e.g., Hovav et al., 2008; Rapp et al., 2010; Hufford et al., 2012; Swanson-Wagner et al., 2012). As with genome-wide SNP screens, these approaches require no a priori assumptions about traits of interest, and hence offer powerful exploratory tools for revealing the effects of domestication at a diversity of biological scales, ranging from DNA sequence through the metabolome to the phenotype.

In this review, we highlight recent insights into the genotype–phenotype connection in crop species and how selection during domestication has shaped phenotypic evolution. We first summarize recent findings across diverse crop species on the molecular genetic basis of domestication-related phenotypes and the nature of the targeted genes and mutational mechanisms. We then discuss recent studies that have examined the effects of selection at biological levels of organization downstream of the coding sequence (e.g., transcriptome, proteome). Finally, as an exploration of parallel evolution among crop species, we examine the genetic basis of changes in flowering time, a trait that has been subject to selection in many crops, to assess the degree to which parallel adaptation has occurred through shared genetic mechanisms.

## Genetic basis of domestication phenotypes

### The domestication syndrome and crop improvement traits

When our ancestors began to shift from collecting wild plants to actively cultivating them, they imposed intense selective pressures for traits that facilitate human cultivation and harvesting of the crop. The resulting phenotypic changes, shared among many food crops, are collectively referred to as the domestication syndrome (Hammer, 1984; Harlan, 1992). Domestication traits in the strict sense may be considered those that distinguish a crop from its wild relatives. For annual cereal crops, which collectively make up the genetically best characterized crop species, traits favored during the initial stages of domestication are generally those that facilitate uniform planting and efficient harvesting. These traits include not only those that are likely to have evolved through conscious selection (e.g., loss of seed shattering, increased yield, decreased chemical, and morphological defenses), but also changes more likely to reflect unconscious selection (e.g., loss of seed dormancy, uniformity in germination and growth phenology, erect growth to facilitate increased plant density in crop fields).

After the initial stages of domestication, cultivated crops have been subject to selection for crop improvement traits (e.g., increased palatability and productivity), and for a diversification in traits that characterize varietal differences (e.g., fruit pigmentation variation, diversification in grain starch composition, and adaptation to different climates and latitudes). While the distinction between domestication traits and later improvement or diversification traits is not always clear (e.g., increased fruit or grain size), the latter traits often may be discerned because they remain variable among different varieties or landraces. Table 1 provides a list of phenotypic changes commonly observed as a result of domestication and crop improvement (see also Harlan, 1992; Miller and Gross, 2011; Meyer et al., 2012).

**Table 1:** **Phenotypic evolution during domestication and improvement of food crops**.

**Wild ancestor**	**Domesticated crop**
**PLANT ARCHITECTURE, MORPHOLOGY, 2° CHEMISTRY**	
Prostrate, spreading growth	Erect, compact plant growth
Axillary branching	Reduced axillary branching
Spines, thorns	Reduced defensive structures
Toxic or unpalatable defense compounds	Reduced toxicity, unpalatability
**PLANT LIFE HISTORY, GROWTH, AND REPRODUCTION**	
Seed dormancy	Reduced seed dormancy
Perennial life history	Annual life history
Sexual reproduction	Asexual/vegetative reproduction
Asynchronous flowering, maturation	Uniform flowering, maturation
Outcrossing	Self-fertilizing
Indeterminate growth	Determinate growth
Photoperiod sensitivity	Reduced or altered photoperiod response: Vernalization requirement Shifted flowering time during growing season
Variable resource allocation	Increased resource allocation to harvested organ (fruit, root, stem, etc.)
**INFLORESCENCE DEVELOPMENT**	
Open inflorescences or panicles	Compact inflorescences/panicles
Many inflorescences, few florets per inflorescence	Reduced inflorescence number, increased florets per inflorescence
Enclosed grains	Exposed, free-threshing grains
**FRUIT AND SEED MORPHOLOGY**	
Dehiscent fruit	Indehiscent fruit
Smaller grains/fruit	Larger grains/fruits or increased number
Spines/Bristles/awns present	Spines/bristles/awns reduced or absent
Uniform morphology	Diversified morphology
**FRUIT AND SEED COMPOSITION**	
Toxic or unpalatable 2° compounds	Reduced or altered defense compounds
Uniform pigmentation	Diversified and/or reduced pigmentation
Uniform carbohydrate composition	Diversified starch and sugar composition

### Genetic changes associated with domestication and crop breeding

As recently as 2006, the number of crop domestication and improvement traits for which the molecular basis was well-understood was just over two dozen (Doebley et al., 2006). Since then, there has been an explosion in studies characterizing domestication-related traits; dozens of genes and causative genetic mutations have now been described, including at least 19 since 2012. Table 2 provides examples of traits and their associated genes that have been molecularly characterized within the last 5 years.

**Table 2:** **Recent examples of functionally characterized genes and mutations that underlie phenotypic changes during crop domestication or improvement**.

**Crop**	**Gene**	**Gene category**	**Trait**	**Causative change**	**Prevalence**	**Gene identification method**	**References**
**PLANT ARCHITECTURE**							
Maize	*tb1 (teosinte branched1)*	Transcriptional regulator	Loss of axillary branches	*cis*-Regulatory via TE insertion	A	QTL mapping	Wang et al., 1999; Studer et al., 2011
Rice	*PROG1*	Transcriptional regulator	Erect growth	AA change (loss of function)	A	QTL mapping	Jin et al., 2008; Tan et al., 2008
Rice	*TAC1*	Unknown (grass specific protein)	Tiller angle (erect growth)	Intron splice site mutation	S	QTL mapping	Yu et al., 2007; Jiang et al., 2012
Rice	*SD1*	Hormone synthesis	Culm length (plant height)	AA changes	S	QTL mapping	Asano et al., 2011
**PLANT GROWTH AND REPRODUCTIVE TIMING**							
**Seed dormancy**							
Rice	*Sdr4*	Transcriptional regulator	Seed dormancy reduction	AA changes	S	QTL mapping	Sugimoto et al., 2010
**Indeterminate vs. determinate growth**							
Common bean	*PvTFL1y*	Transcriptional regulator	Determinate growth	TE insertion, gene deletion, AA change, indels, splice site mutation	S	QTL mapping, association mapping	Kwak et al., 2012; Repinski et al., 2012
Soybean	*Dt1 (GmTfl1)*	Transcriptional regulator	Determinate growth	AA change	S	Candidate gene	Tian et al., 2010
**Vernalization requirement for flowering**							
Barley	*HvCEN*	phosphatidyl ethanolamine–binding protein (PEBP); homolog of Antirrhinum *CENTRORADIALIS (CEN)*	Loss of vernalization requirement; altered photoperiod response allows spring growth habit in northern latitudes	AA change	S	GWAS, mutant analysis	Comadran et al., 2012
Barley	*Ppd-H1*	Pseudoresponse regulator (PRR) protein; circadian clock component, affects timing of expression floral regulatory activators	Loss of vernalization requirement; altered photoperiod response allows spring growth habit in northern latitudes	AA change	S	QTL mapping, association mapping	Turner et al., 2005; Jones et al., 2008
Rapeseed	*BnFLC.A10*	Transcriptional regulator; ortholog of *Arabidopsis FLC*, vernalization-mediated repressor of floral induction	Photoperiod sensitivity; increased gene expression confers vernalization requirement, allowing for winter growth habit	*cis*-regulatory via TE insertion	S	QTL mapping	Hou et al., 2012
Wheat	*Vrn1*	Transcriptional regulator; putative wheat *APETALA1*, floral induction integrator	Photoperiod sensitivity: loss of function alleles generate vernalization requirement	*cis*-regulatory, including TE-mediated promoter duplication	S	QTL mapping	Yan et al., 2003; Golovnina et al., 2010
Wheat	*Vrn2 (ZCCT1 and ZCCT2)*	Transcriptional regulator, CCT domain protein; closest rice homolog is *Ghd7*; repressor of floral induction; not a homolog of *Arabidopsis VRN2*	Photoperiod sensitivity: loss of function alleles eliminate vernalization requirement, allowing for spring wheat	AA change and gene deletions	S	QTL mapping	Yan et al., 2004; Distelfeld et al., 2009
**Flowering time during growing season**							
Lentil	*SN* (*ELF3*)	Substrate adaptor protein; ortholog of *Arabidopsis EARLY FLOWERING 3*, coordinates circadian clock function	LD photoperiod sensitivity: loss of function allows for spring flowering without LD conditions	Splice site mutation, premature stop	S	QTL mapping, candidate gene	Weller et al., 2012
Maize	*ZmCCT*	Transcriptional regulator; CCT domain protein, homolog of rice photoperiod response regulator *Ghd7*, represses floral induction	SD photoperiod sensitivity: reduced function allows earlier flowering under LD conditions in temperate varieties	Not definitively determined	S	NAM, candidate gene	Hung et al., 2012
Pea	*HR* (*ELF3*)	Substrate adaptor protein; ortholog of *Arabidopsis EARLY FLOWERING 3*, coordinates circadian clock function	LD photoperiod sensitivity: loss of function allows for spring flowering without LD conditions	Frameshift insertion, premature stop	S	QTL mapping, candidate gene	Weller et al., 2012
Rice	*DTH2*	Transcriptional regulator; CONSTANS-like protein, mediates photoperiod-regulated flowering response independent of *Hd1* and *Ehd1*	Photoperiod sensitivity: altered function allows earlier flowering under LD conditions of higher latitudes (minor-effect QTL)	AA changes	S	QTL mapping	Wu et al., 2013
Rice	*Ehd1*	Transcriptional regulator; B-type response regulator, promotes flowering; no clear *Arabidopsis* ortholog	Photoperiod sensitivity: promotes SD flowering in the absence of functional *Hd1*; loss of function leads to later flowering in the absence of functional *Hd1*	Premature stop (transposon insertion), AA change	S	QTL mapping	Doi et al., 2004; Saito et al., 2009
Rice	*Ghd7*	Transcriptional regulator; CCT domain protein, represses *Ehd1* under LD photoperiod	LD photoperiod response (also grain number, plant height): reduced function allows earlier flowering under LD conditions in short-season climates	AA changes, premature stop, gene deletion, *cis*-regulatory	S	QTL mapping	Xue et al., 2008; Lu et al., 2012
Rice	*Hd1*	Transcriptional regulator; ortholog of *Arabidopsis CONSTANS*	SD and LD photoperiod sensitivity: loss of function leads to later flowering under SD conditions, earlier under LD conditions	Frameshift mutations, TE frameshift insertions; premature stop codons	S	QTL mapping	Yano et al., 2000; Takahashi et al., 2009; Fujino et al., 2010; Ebana et al., 2011
Rice	*Hd6*	Protein kinase; α-subunit of casein kinase II (CK2); functions in *Hd1*-mediated suppression of *Hd3a* under LD photoperiod	Photoperiod sensitivity (limited subset of temperate japonica varieties): loss of function leads to earlier flowering under LD conditions	Premature stop codon	S	QTL mapping	Takahashi et al., 2001; Yamane et al., 2009; Ogiso et al., 2010
Rice	*Hd17*	Transcriptional regulator; homolog of *Arabidopsis EARLY FLOWERING 3* (*ELF3*), derived allele downregulates floral repressor *Ghd7*	Photoperiod sensitivity: derived allele leads to earlier flowering under LD conditions	AA change	S	QTL mapping	Matsubara et al., 2008, 2012
Sorghum	*Ma1* (*SbPRR37*)	Pseudoresponse regulator (PRR) protein; regulatory repressor in photoperiod-mediated flowering induction	Photoperiod sensitivity: loss of function allows flowering in LD temperate climates	Frameshift deletion, premature stop, AA change	S	QTL mapping, candidate gene	Murphy et al., 2011
Soybean	*E1*	Putative transcriptional regulator; related to B3 domain plant proteins, potentially functions in phytochrome A light signaling pathway	Photoperiod sensitivity: loss or reduction in SD flowering response allows earlier flowering in high latitude, short-season climates	Frameshift deletion, gene deletion, AA change	S	QTL mapping	Xia et al., 2012
Sunflower	*HaFT1* (and paralogs)	Transcriptional regulator; ortholog of *Arabidopsis FLOWERING LOCUS T*, floral induction integrator	Photoperiod response: shifts to later and earlier flowering under LD conditions	Frameshift (altered but functional protein)	A	Candidate gene, QTL mapping	Blackman et al., 2010
**Tuberization timing**							
Potato	*StCDF1*	Transcriptional regulator; DOF (DNA-binding with one finger) transcription factor, mediates between circadian clock and tuberization signaling	SD photoperiod sensitivity: loss of function alleles allow tuberization under LD temperate growing conditions	Insertions create truncated proteins	S	QTL mapping	Kloosterman et al., 2013
**INFLORESCENCE DEVELOPMENT**							
Barley	*Vrs1*	Transcriptional regulator	Inflorescence architecture (2- vs. 6-rowed)	Premature stop (insertion, deletion, or AA change)	S	QTL mapping	Komatsuda et al., 2007; Sakuma et al., 2013
Barley	*INT-C (HvTB1)*	Transcriptional regulator	Inflorescence architecture (2- vs. 6-rowed)	Not definitively identified	S	GWAS using population sample	Ramsay et al., 2011
Barley	*Nud*	Transcriptional regulator	Naked (free-threshing) grains	Chromosomal deletion	S	QTL mapping	Taketa et al., 2008
Maize	*ra1 (ramosa1)*	Transcriptional regulator	Inflorescence architecture	Not definitively identified (likely *cis*-regulatory)	A	Candidate gene	Sigmon and Vollbrecht, 2010
Maize	*FEA2*	LRR receptor-like protein	Kernel row number	Not definitively identified (likely *cis*-regulatory)	S	QTL mapping, Mutant screens	Bommert et al., 2013
Rice	*OsLG1 (SPR3)*	Transcriptional regulator	Closed panicle (outcrossing rate and seed shattering)	Probably *cis*-regulatory	A (not definitively confirmed)	QTL mapping	Ishii et al., 2013
Sorghum	*Sh1* (*Shattering1*)	Transcriptional regulator	Shattering	*cis*-regulatory and deletion	A	QTL mapping	Lin et al., 2012
Wheat	*Q* and homeologs	Transcriptional regulator	Free-threshing and other traits	*cis*-regulatory and AA change	S	Mapping in deletion lines, candidate gene analysis	Simons et al., 2006; Zhang et al., 2011
**FRUIT AND SEED MORPHOLOGY**							
Rice	*GS5*	Putative positive regulator of mitosis	Grain size	*cis*-regulatory	S	QTL mapping	Li et al., 2011
Rice	*GIF1*	Cell wall invertase	Grain filling	Probably *cis*-regulatory	A	Mutant screens, QTL mapping	Wang et al., 2008
Rice	*GS3*	Putative negative regulator of ovule development	Grain size and length	Premature stop	S	QTL mapping	Fan et al., 2006; Takano-Kai et al., 2009
Rice	*qSW5*	Putative regulator of outer glume development	Grain width	Deletion	S	QTL mapping	Shomura et al., 2008
Rice	*GW2*	ubiquitin ligase (putative repressor of cell division)	Grain width and weight	Premature stop (deletion)	S (survey incomplete)	QTL mapping	Song et al., 2007
Rice	*OsPPKL1*	Putative protein phosphatase	Grain length	AA change	S	QTL mapping	Zhang et al., 2012b
Rice	*OsSPL16* (*GW8)*	Transcriptional regulator	Grain shape and size	*cis*-regulatory	S	QTL mapping	Wang et al., 2012
Tomato	*fas* (*fasciated*)	Transcriptional regulator	Locule number (fruit size)	*cis*-regulatory	S	QTL mapping	Cong et al., 2008
Tomato	*lc*	Not definitively identified (*WUSCHEL* or *WD40)*	Locule number (fruit size)	*cis*-regulatory	S	QTL mapping, association mapping	Muños et al., 2011; Ranc et al., 2012
Tomato	*SUN*	Positive growth regulator	Elongated fruit shape	TE-mediated gene duplication	S	QTL mapping	Xiao et al., 2008; Rodríguez et al., 2011
**PHYSIOLOGICAL ADAPTATION**							
Rice	*PSTOL1*	Protein kinase	Phosphorous deficiency tolerance	Gene presence/absence	S	QTL mapping	Gamuyao et al., 2012
**FRUIT AND SEED COMPOSITION**							
Amaranths (3 species)	*Waxy (GBSS1)*	Enzyme (starch synthase)	Starch (glutinous phenotype)	Premature stop codons	S	Candidate gene	Park et al., 2010
Broomcorn millet	*GBSS1 (2 genes) (Waxy)*	Enzyme (starch synthase)	Starch (glutinous phenotype)	Indels and AA change	S	Candidate gene	Hunt et al., 2010, 2013
Citrus (orange)	*Ruby*	Transcriptional regulator	Anthocyanin production (blood orange)	TE insertion in *cis*-regulatory region	S	Candidate gene	Butelli et al., 2012
Citrus species	*Cm1,2RhaT* and *Cm1,6RhaT*	Enzymes (rhamnosyltransferases)	bitterness	Frameshift mutations and gene absence	S	Candidate gene	Frydman et al., 2012
Grape	*VvMybA* gene family	Transcriptional regulator	Berry color	TE insertion and AA change	S	Candidate gene	Walker et al., 2007; Fournier-Level et al., 2010
Maize	*GBSS1 (Waxy)*	Enzyme (starch synthase)	Starch (glutinous phenotype)	Deletions	S	Candidate gene	Fan et al., 2008, 2009
Rice	*BADH2*	Enzyme (betaine aldehyde dehydrogenase)	Fragrance	Premature stop (deletion or AA change)	S	QTL mapping	Bradbury et al., 2005; Kovach et al., 2009; Shao et al., 2012
Rice	*Bh4*	Amino acid transport protein	Hull color	Deletions and premature stop codon	S	QTL mapping	Zhu et al., 2011
Rice	*Phr1*	Enzyme (polyphenol oxidase)	Grain discoloration (oxidation)	Premature stop (insertion or deletion)	S	QTL mapping	Yu et al., 2008; Gross et al., 2009
Sorghum	*Tannin1*	WD40 protein (coordinates multiprotein complexes)	Grain pigmentation	Frameshifts causing premature stop codons	S	QTL mapping	Wu et al., 2012

Traits altered through modern crop breeding (20th Century or later) are not included. For the category of prevalence, “A” indicates that the genetic change is characteristic of all domesticates, and “S” indicates that the change is present in a subset of domesticated varieties. For flowering time genes, details are provided on homology to other flowering time genes; LD and SD indicate long-day and short-day photoperiod, respectively.

A comparison of Tables 1 and 2 reveals some of the current limitations and biases in our understanding of the genetics of domestication phenotypes. First, the vast majority of genes characterized to date have been identified at least partly through trait mapping using advanced generation mapping populations—primarily biparental QTL mapping populations (see Table 2). Nearly all such mapping populations are created using species where at least one generation can be produced per year. A consequence is that our inferences presently are limited almost entirely to traits in sexually propagated, annual crop species; indeed, all but two of the crops in Table 2 (grapes and citrus) are grown as annuals, and the majority of these are cereal crops. Reliance on biparental mapping populations has also predisposed inferences toward the identification of a relatively few QTLs of large effect, since only the genetic variation present in the two parental lines is represented in the mapping population. As association studies now begin to make use of more genetically diverse mapping populations (e.g., NAM, GWAS), it is becoming increasingly clear that, as with complex traits in wild species, the genetic architecture of many domestication-related traits involves many genes with small effects (see, e.g., Buckler et al., 2009; Kump et al., 2011; Poland et al., 2011; Tian et al., 2011; Zhao et al., 2011; Cook et al., 2012; Huang et al., 2012b).

An additional bias evident in Table 2 is toward traits where obvious candidate genes are already known based on previous research in model organisms. Knowledge of candidate genes facilitates identification of the causative gene within a genomic region containing a QTL peak or selection signature. For example, the *Arabidopsis* flowering time pathway is among the best characterized developmental pathways in plants, providing clear candidate genes for studies of selection on flowering time. Correspondingly, nearly one-third of the examples in Table 2 involve changes in photoperiod response or other aspects of flowering time. Keeping in mind the caveats that the taxa, traits, and genes in Table 2 are not representative of all crops and domestication phenotypes, we explore below what might be inferred about the nature of molecular changes during domestication and crop improvement.

#### Genetic targets of selection

In their 2006 review of the molecular genetics of crop domestication, Doebley and colleagues noted that changes in developmentally or morphologically complex phenotypes typically involve selection on genes that encode transcriptional regulators, as opposed to genes encoding structural proteins or enzymes. This observation has continued to hold true for domestication-related traits, and it reflects a pattern that appears to be more generally characteristic of morphological evolution (Brakefield, 2011; De Bruijn et al., 2012). For those phenotypic categories in Table 2 that involve changes in complex morphological traits or developmental processes (plant architecture, plant growth and reproductive timing, inflorescence development, and fruit and seed morphology), nearly all characterized genes encode either transcriptional regulators or other proteins that function in regulating basic developmental processes, such as cell growth and division (e.g., rice *GS5, GIF1*, and *GW2*, all controlling grain development) or hormone synthesis (e.g., rice *SD1*, controlling plant height). In contrast, traits that involve specific metabolic pathways, such as carbohydrate or pigment synthesis, may arise either through selection on regulatory genes (e.g., grape and orange fruit pigmentation; Walker et al., 2007; Fournier-Level et al., 2010; Butelli et al., 2012) or through selection on structural genes in the pathway. For example, in several different grain crops there has been selection for glutinous (waxy) varieties, which lack the starch amylose (e.g., rice, maize, barley, sorghum, millets, and amaranths). In all of these cases, the glutinous phenotype has arisen through selection for loss-of-function mutations at the *Waxy* (*GBSS1*) locus, which encodes the starch synthase required for amylose synthesis (see Table 2 for recent examples).

#### Mutational mechanisms

Evolution is fundamentally opportunistic with respect to its causative mechanisms. It is perhaps unsurprising, then, that Table 2 reveals a wide diversity in the nature of the mutations that underlie domestication-related phenotypes. These include *cis*-regulatory mutations that increase gene expression (e.g., rapeseed *BnFLC.A10*, controlling vernalization; Hou et al., 2012), and mutations in coding regions that result in modified proteins that remain functional but have altered activity (e.g., sunflower *HaFT1*, controlling flowering time; Blackman et al., 2010). The largest proportion of these domestication-related genetic changes, however, are those that result in a loss of gene function. Common mechanisms by which this occurs include: SNPs in coding regions that generate premature stop codons (e.g., rice *GS3*, controlling grain length; Takano-Kai et al., 2009); indels in coding regions that create frameshift mutations (e.g., pea *HR*, controlling photoperiod sensitivity; Weller et al., 2012); amino acid replacements that lead to a loss of protein function (e.g., rice *PROG1*, controlling erect plant growth; Jin et al., 2008; Tan et al., 2008); intron splice site mutations (e.g., rice *TAC1*, controlling tiller angle; Yu et al., 2007); and indels or SNPs in *cis*-regulatory regions that disrupt transcription (e.g., grape *VvMybA1*, controlling fruit pigmentation; Lijavetzky et al., 2006; Fournier-Level et al., 2010). The apparent prevalence of loss of function mutations in crop plants likely reflects the fact that coding sequences and their promoters provide relatively large mutational targets. Loss of function mutations might also be differentially tolerated in agricultural settings, if their negative pleiotropic consequences are sufficiently offset by the desirability of a domestication-related trait. In addition, since loss of function mutations occur more frequently than those leading to a gain in function, they would be expected to represent a greater proportion of the mutations that have arisen since the origins of agriculture.

Insofar as mutational mechanisms are concerned, two key processes are implicated as especially significant sources of genetic variation in crop plants, namely, transposable element (TE) activity (a portion of which also entail loss-of-function mutations) and gene duplication events. We consider each of these separately below for crop domestication phenotypes.

***Transposable elements.*** TE proliferation has played a major role in shaping the evolution of plant genomes, including crop species. A recent review indicates that TEs constitute between 22% and 85% of the genomes of 11 crop species examined (Morrell et al., 2011). The replication and proliferation of TEs not only affects genome size and structure, but it can also have major phenotypic consequences—either directly through TE insertions into genes and their *cis*-regulatory regions, or through genomic structural changes such as gene duplications and chromosomal rearrangements that alter levels of gene expression. Consistent with this mutational capacity, a number of TE-mediated mutations have been documented to underlie domestication-related traits. This includes the causative mutation at what is perhaps the most celebrated of all domestication loci, the maize locus *tb1*. Domesticated maize (*Zea mays* subsp. *mays*) differs from its wild ancestor, teosinte (*Zea mays* subsp. *parviglumis*), in lacking axillary branch development; this change is largely due to increased expression of *tb1*, a transcriptional regulator that represses growth (Doebley et al., 1997). Through a combination of fine-mapping in maize-teosinte introgression lines and selection screens in a diverse germplasm panel, Doebley and colleagues demonstrated that *tb1* upregulation in the crop results from the insertion of a *Hopscotch* retroelement into the gene's *cis*-regulatory region, which, remarkably, is located ~60 kb upstream of the coding region (Studer et al., 2011). Molecular dating of this TE insertion suggests that it predates the time frame of maize domestication and so likely existed as standing genetic variation in teosinte prior to selection by humans.

Fruit characteristics provide other well-documented examples of human selection on TE-mediated mutations. In grapes, non-pigmented (“white”) berry color arises through the absence of anthocyanin synthesis during fruit development. The modern white grape phenotype appears to have arisen through sequential selection for loss-of-function mutations in two adjacent anthocyanin regulatory genes: first, a single-nucleotide non-synonymous mutation in *VvMYBA2*, and then a *Gret1* gypsy-type retrotransposon insertion in the promoter of *VvMYBA1* (Kobayashi et al., 2005; Walker et al., 2007; Fournier-Level et al., 2010). In domesticated tomato, the elongated fruit shape found in some heirloom varieties is attributable to a *Copia*-like retrotransposon-mediated duplication of the *SUN* gene; the duplicated gene copy is positioned so that it is under *cis*-regulatory control of a different gene (encoding a defensin protein) that is expressed at high levels during fruit development (Xiao et al., 2008). In oranges, the anthocyanin production that produces the blood orange phenotype occurs through the activity of another *Copia*-like retrotransposon. In this case, exposure of ripening fruits to cold induces retrotransposon-mediated transcriptional activation of *Ruby*, a *Myb* regulatory gene in the anthocyanin synthesis pathway (Butelli et al., 2012). Other domestication-related traits that have evolved through TE activity include determinate growth in common bean (*PvTFL1y*; Repinski et al., 2012); vernalization requirement in rapeseed (*BnFLC.A10*; Hou et al., 2012) and wheat (*Vrn1*; Golovnina et al., 2010); and photoperiod sensitivity in rice (*Ehd1*; Saito et al., 2009).

***Gene duplication.*** The tomato fruit shape example above illustrates the potential for TE-mediated gene duplications to alter crop phenotypes. More generally, gene duplication is a prominent feature of plant genome evolution, reflecting a history that includes repeated episodes of whole-genome doubling (Jiao et al., 2011), as well as other duplication processes including TE activity, unequal crossing over, and other chromosomal structural aberrations (Flagel and Wendel, 2009). Accordingly, nearly all genes in modern plant genomes exist as members of small to large multigene families, with paralogous gene copies sharing various degrees of relatedness as a function of the amount of time elapsed since duplication (and with some paralogs predating the origin of seed plants) (Jiao et al., 2011).

In the context of crop domestication, a wealth of indirect evidence suggests a role for polyploidization in generating adaptive plasticity and novel phenotypic variation for domestication-related traits [reviewed by Paterson (2005); Udall and Wendel (2006)]. Some of the clearest evidence is found in the grain hardness and free-threshing phenotypes of hexaploid bread wheat, a crop that originated through hybridization of a tetraploid wheat (containing the A and B ancestral diploid genomes) and a diploid (contributing the D genome). Grain hardness has been subject to diversifying selection in wheat, with hard grains favored in the tetraploid wheats grown for pasta, soft grains favored for bread flour, and semi-hard grains favored in some bread wheat varieties. The trait is controlled by the complex hardness locus (*Ha*), which was present in all three ancestral diploid genomes, and which generates the soft wheat phenotype when functional. Deletions of *Ha* from both of the ancestral genomes of tetraploid wheat created the hard grain phenotype, and the ancestral soft grain phenotype was restored in bread wheat by the contribution of the D-genome *Ha* locus; subsequent selection for deletions and complex rearrangements in the D-genome locus gave rise to the semihard hexaploid wheats (Chantret et al., 2004). The wheat *Q* locus, which controls the free-threshing phenotype and other aspects of plant and inflorescence development, has an even more complex history (Zhang et al., 2011). In this case, free-threshing grains originated through a combination of ancient gene duplications within the ancestral diploid genomes, loss of alternate paralogs in the different genomes, post-polyploidization selection for a single amino acid replacement in the A-genome homeolog, pseudogenization of the B-genome homeolog (but with continued transcription, contributing to expression regulation of the other homeologs), and subfunctionalization of the D-genome homeolog.

In other cases, gene duplications unrelated to polyploidization have played a role in crop domestication phenotypes. The TE-mediated duplication of the tomato *SUN* gene described above provides one such example. Another well documented instance involves paralogous copies of the flowering time gene *FT* (*FLOWERING LOCUS T)* in sunflower and selection for changes in flowering time during domestication and later crop improvement (Blackman et al., 2010, 2011). *FT* genes function as positive regulators of reproductive meristem development, and Blackman and colleagues were able to identify four paralogous copies in sunflower, three of which appear to be functional and show evidence of having played a role in a shift toward later flowering time during sunflower domestication. Specifically, the paralogs show divergence in expression patterns between wild and domesticated sunflowers, co-localization with flowering time QTLs, and molecular signatures consistent with selection during domestication. In addition, one of the paralogs, *HaFT1*, shows evidence of selection for a protein-coding frameshift mutation that alters floral developmental timing through interference with the expression of another paralog *HaFT4* (see Table 2). Interestingly, modern commercial sunflower varieties have been selected for early flowering, the opposite direction as was favored during domestication. The ability of the crop to respond to these contrasting selective pressures may have been facilitated by the partial functional redundancy conferred by the presence of multiple of *FT* paralogs [see discussion by Blackman et al. (2011)].

## Between genotype and phenotype

Given the rapid expansion in the application of high-throughput technologies to the study of crop plant evolution, we anticipate that the pace of discovery of the underpinnings of domestication and improvement traits will continue to increase. In addition to enriching our understanding of the spectrum and relative frequency of causal mutations and the underlying genetic architecture of specific traits, the increasing application of genome-scale systems biology approaches promises to shed qualitatively new light on crop plant evolution. The simultaneous analysis of multiple “omics” (e.g., genomics, transcriptomics, proteomics, metabolomics) in combination with analyses of pathways and networks across various scales (temporal, developmental) offer new opportunities to reveal the intricacies of domestication and crop improvement, and by extension (echoing the words of Darwin invoked in the introduction to this review), the evolutionary process in general. Much of this review has focused on the mutations responsible for phenotypes found in crop plants, and to be sure, considerable progress has been made in this regard (Table 2). But there is a vast biology lying between genotype and phenotype, with the latter reflecting the end product of a complex transduction and propagation from genotype through the transcriptomic, proteomic, and metabolomic networks that lead to biosynthesis and, ultimately, to phenotype.

Recent studies in this area have provided clues into the types of complexity we might expect. Working in maize, Hufford et al. (2012) combined genome resequencing with comparative expression profiling and found a surprisingly large number of genomic regions that may have been targets of selection during domestication (484 regions) and crop improvement (695 regions). Candidate domestication genes show greater changes in gene expression between maize and teosinte than do non-candidate genes, are on average expressed at higher levels, and have reduced expression variability; the latter is interpreted as potentially reflecting directional selection for a reduction in *cis*-regulatory variation. An extension of this work (Swanson-Wagner et al., 2012) used comparative expression profiling of seedlings in 24 teosinte and 38 maize accessions; many of the 600 differentially expressed genes occur in genomic locations that were identified in population genomic diversity screens as potential targets of selection (Hufford et al., 2012).

Studies in cotton also reveal evidence of large-scale rewiring of the transcriptome in response to domestication. Rapp et al. (2010) studied the transcriptome of developing cotton (*Gossypium hirsutum*) “fibers” (seed epidermal trichomes) in both wild and domesticated cotton during five stages representing primary and secondary wall synthesis. They detected significantly altered expression for 9645 genes, or about 25% of the genes in the genome. This is especially remarkable, not just because of the high level of “transcriptomic rewiring” that this reflects, but also because it is observed for a single-celled structure. Other transcriptomic studies in the cotton model system are revealing a comparable, massive rewiring of the transcriptome accompanying domestication (Chaudhary et al., 2008, 2009; Yoo et al., unpublished).

We will also likely soon see the fruitful extension of these types of analyses to levels beyond the transcriptome, toward an understanding of how the transcriptional network propagates through the proteome and beyond to condition new phenotypes. A recent example is provided by Hu et al. (2013), who used advanced proteomic profiling tools in an elite cotton cultivar and a wild accession to gain insight into cotton fiber development and evolution. Using iTRAQ LC-MS/MS technology, they identified ~1000 different proteins in fiber cells, of which about 20% showed differential expression between wild and cultivated forms. A key observation was that human selection appears to have shifted the timing of developmental modules, such that some of these occur earlier in domesticated than in wild cotton.

The results of Hu et al. (2013) demonstrate the power of complementary transcriptomic and proteomic approaches for the study of the domestication process. This also is exemplified by a second study in cotton (Bao et al., 2011), where genomic and proteomic tools were used to investigate one of the protein families (profilin) implicated as highly up-regulated during cotton domestication. Rather than occurring through upregulation of a single profilin gene, all five of the profilin genes expressed in cotton fibers were simultaneously up-regulated. This pattern presumably reflects the downstream effects of upstream regulatory alterations (or potentially just a single mutation) whose effects are propagated through the system during cellular development to affect transcriptome and proteome levels for the entire profilin gene family.

An exciting prospect for the future will be to begin to dissect or partition the complexity that underlies the evolutionary transformation of phenotypes into its constituent parts, so that we can begin to appreciate the effects of and interrelationships among these components on the various “omics” that lie between genotype and phenotype (Mackay et al., 2009). Progress in this direction will almost assuredly emerge from the simultaneous utilization of the tools of systems biology, combined with more traditional QTL analyses and other advanced breeding populations, such as introgression lines. As an example of this systems approach, one can envision multidimensional omics and computional comparisons among near-isogenic introgression lines that have been generated between wild and domesticated populations of a given crop plant.

## Parallel evolution in domesticated crops

Unlike in earlier decades, when “parallelism” or “convergence” (Arendt and Reznick, 2008) were limited to observations at the morphological level, modern technologies permit the analysis of these phenomena at multiple biological scales. In the context of crop domestication, parallel responses to selection have been studied both at the level of gene expression and at the level of underlying mutational changes. Recent studies in cotton serve to illustrate the types of parallel changes that may be observed with respect to gene expression. Within the genus *Gossypium*, three independent domestications occurred, involving two allopolyploids (*G. barbadense* and *G. hirsutum*, the latter of which constitutes ~90% of the world cotton crop) and one diploid (*G. herbaceum*). In their study of the profilin gene family described above, Bao et al. (2011) were able to document that upregulation of the entire profilin gene family has occurred not only with the domestication of *G. hirsutum*, but also in parallel in the two other domestication events. In another study of cotton fiber development, Chaudhary et al. (2009) examined changes in a class of genes implicated as developmentally important for their roles in fine-tuning cellular redox levels (reactive oxygen species, or ROS genes), which are important for cell expansion; they discovered that several antioxidant genes were substantially up-regulated in the three domesticated forms of cotton, in comparison to their wild antecedents. Remarkably, many of the ROS-related processes diagnosed as possible targets of selection were shared among the diploid and allopolyploid cultigens, but involved different sets of antioxidant genes. This finding suggests that selection may have operated to achieve similar ends by different underlying genetic mechanisms. It will be of considerable interest to elucidate and compare the specific genomic changes in each of the cotton species that have mediated these parallel responses to domestication.

### The genetic basis of parallel evolution: flowering time as an example

As discussed earlier in this review, much of what we currently understand about the mutational basis of domestication-related phenotypic evolution comes from traits and genes related to flowering time. Shifts in the timing of reproduction generally are not associated with the initial stages of domestication; rather, these changes have occurred through later diversifying selection, as domesticated crop varieties were introduced into latitudes and climates where the native-range flowering time response would be suboptimal or maladaptive, or for the development of earlier maturing varieties in the native range. For rice, maize, sorghum, cotton, and other crops that were domesticated in tropical and near-tropical regions, flowering is often promoted under short-day (SD) photoperiod. The spread of these crops into temperate regions was enabled by selection for reduced or altered SD response, allowing them to be cultivated in the long-day (LD) summer growing seasons of higher latitudes (e.g., Murphy et al., 2011; Hung et al., 2012; Matsubara et al., 2012). Similarly, cultivation of the potato in temperate latitudes was only possible with selection for varieties lacking a SD tuberization response (Murphy et al., 2011). In the case of some temperate crops, including peas and lentils, reproduction occurs under long days in the native range (the Mediterranean and Fertile Crescent in the case of these pulses), and selection against LD photoperiod response allowed for emergence of the spring-flowering varieties grown at higher latitudes (Weller et al., 2012).

Other temperate crops have undergone selection to either lose or acquire a vernalization requirement, whereby flowering occurs only following exposure to an extended period of cold. Crop varieties possessing a vernalization requirement have a winter growth habit, germinating in the fall and increasing biomass throughout the winter months before flowering in the spring. This growth habit is adaptive in climates with mild winters and hot, dry summers that are inhospitable for growth. Selection for the acquisition of a vernalization requirement has occurred in winter wheat varieties (Yan et al., 2003) and some varieties of rapeseed (Hou et al., 2012). In contrast, selection for a loss of vernalization requirement has occurred in spring barley (e.g., Turner et al., 2005; Comadran et al., 2012) and spring wheat (Yan et al., 2004), where the spring growth habit allows planting and harvesting in northern latitudes with short growing seasons.

In considering changes in flowering time as a case study of parallel evolution in crops, it is important to recognize that not all crops have been subject to selection for the same changes, nor do all crops share identical developmental genetic components of the flowering time pathway. For example, whereas selection on vernalization requirements has played a major role in the development of winter and spring varieties of temperate cereals such as wheat and barley, the vernalization signaling pathway is absent in tropical cereals such as rice, maize, and sorghum. Thus, selection on flowering time in the tropical grasses is restricted to shifts toward earlier or later flowering within the active growing season, as opposed to selection for spring vs. winter growth habit. It is also important to note that identifying orthologous flowering time genes in two species does not guarantee that they actually share the same function. For example, the *Arabidopsis* photoperiod pathway gene *CONSTANS* (*CO*) functions as an activator of downstream floral pathway integrators under LD photoperiod, while its rice ortholog, *Hd1*, has the opposite effect, repressing downstream genes under LD photoperiod and activating them in SD conditions (Tsuji et al., 2011).

**Figure 1:**
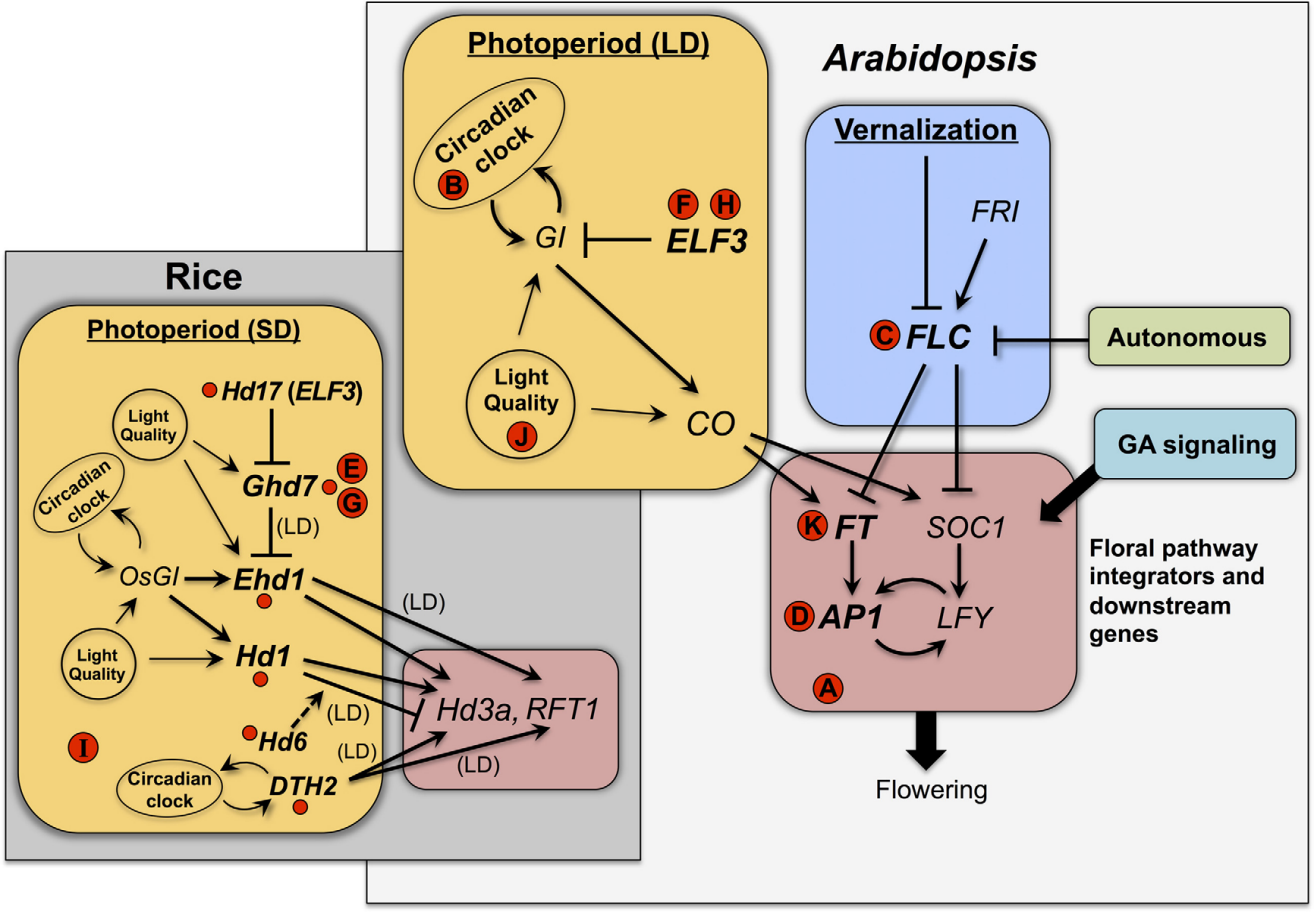
**Flowering time genes recently identified as targets of selection in crop species**. The *Arabidopsis* flowering time pathway is represented as a simplified schematic based on Ballerini and Kramer (2011) and references in Table 2; photoperiod response occurs under long-day (LD) conditions. The gray inset shows a simplified schematic of the rice photoperiod response pathway and immediate downstream genes (*Hd3a* and *RFT1*, both *Arabidopsis FT* homologs), based on Tsuji et al. (2011) and references in Table 2. Small red circles correspond to rice flowering time genes in Table 2, with known regulatory interactions among them indicated by arrows and lines; interactions are for short-day (SD) photoperiod unless long-day (LD) is indicated. Letters in red circles correspond to homologous genes from other crop species, as follows: **(A)** barley *HvCEN*, **(B)** barley *Ppd-H1*, **(C)** rapeseed *BnFLC.A10*, **(D)** wheat *Vrn1*, **(E)** wheat *Vrn2*, **(F)** lentil *SN*, **(G)** maize *ZmCCT*, **(H)** pea *HR*, **(I)** sorghum *Ma1*, **(J)** soybean *E1*, **(K)** sunflower *HaFT1* and paralogs. Positions of letters indicate known homologies to *Arabidopsis* or rice genes.

Depending on the crop species and the nature of its flowering time response, changes in flowering time could potentially arise from either decreased or increased expression of diverse regulatory factors in the flowering time pathway [see reviews by Ehrenreich et al. (2009); Andrés and Coupland (2012); Matsoukas et al. (2012)]. Figure 1 provides a schematic representing the portions of the *Arabidopsis* and rice flowering time pathways that contain genes or gene homologs that have been targets of selection in crop species. Potential targets of selection could include: (1) genes that mediate photoperiod and related circadian clock functions [e.g., orthologs of *Arabidopsis EARLY FLOWERING* 3 (*ELF3*) and *CONSTANS* (*CO*)]; (2) those that mediate vernalization cues [e.g., orthologs of *Arabidopsis FLOWERING LOCUS C* (*FLC*)]; (3) those involved in other components of floral induction signaling (e.g., the autonomous and gibberellin (GA) signaling pathways); and (4) those that function further downstream in the flowering pathway as integrators of the different signaling pathways [e.g., orthologs of *FLOWERING LOCUS T* (*FT*) and *APETALA1* (*AP1*)]. Among the domestication-related flowering time genes that have been molecularly characterized in recent years (Table 2), most involve some aspect of photoperiod response, either directly involving photoperiod and circadian clock signaling (e.g., *ELF3* in legumes and rice; (Matsubara et al., 2012; Weller et al., 2012)) or involving downstream floral pathway integrators (e.g., *FT* and *AP1* homologs in sunflower and wheat, respectively; Yan et al., 2003; Blackman et al., 2010) (Figure 1).

At least seventeen domestication-related flowering time genes have been molecularly characterized in the last 5 years (Table 2), not counting genes that were subject to selection in the 20th century (e.g., rice *Hd5*; Fujino et al., 2013). More than one-third of these come from the genomic model species rice, but other cereals (maize, sorghum), legumes (pea, lentil, soybean), and sunflower are also represented. Some sharing of genetic targets of selection is evident across these crops (Figure 1). For example, mutations in the photoperiod pathway gene *ELF3* have played a role in the emergence of early flowering varieties of peas, lentils, and rice (Matsubara et al., 2012; Weller et al., 2012). Similarly, homologs of the downstream photoperiod regulator *Ghd7* were targets of selection in both rice and maize for earlier flowering under LD photoperiod (Xue et al., 2008; Hung et al., 2012), and in wheat for spring growth habit (Distelfeld et al., 2009). On the other hand, it is equally noteworthy that a diverse number of different flowering time genes have been identified to date as targets of selection in crops. These include at least six different photoperiod response genes in rice alone, as well as orthologs of the well-characterized Arabidopsis genes *FLC* (rapeseed *BnFLC.A10*; Hou et al., 2012), *FT* (sunflower *HaFT1* and paralogs; Blackman et al., 2011) and *AP1* (wheat *Vrn1*; Yan et al., 2003) (Figure 1). While flowering time is admittedly a single trait and the studies published to date provide a relatively small sample size, these findings potentially suggest a wide breadth in the number and types of genes that may serve as targets of selection conditioning a common phenotype in a diversity of species. The underlying mutational mechanisms are similarly diverse, involving a variety of *cis*-regulatory and protein-coding changes (Table 2).

## Conclusions

It is an exciting time in evolutionary biology, one characterized by unprecedented experimental power. The application of a suite of advanced technologies to domestication-related traits in multiple crop-wild plant model systems is rapidly increasing our ability to discover the genes affected by human selection during both initial domestication and subsequent phases of crop improvement. This collective effort is providing a rich comparative database of the mutational spectra underlying crop evolution and the manner in which changes at the genetic level propagate through various levels of organization to confer the targeted phenotypes. As the number of molecularly characterized traits continues to grow, it will become increasingly possible to capitalize on these traits and genes to study how the broader developmental context in which they function has shaped the evolutionary process. It is likely that the most exciting and novel insights will derive from the utilization of multiple “omics” tools in an integrative framework, all brought to bear on specific genotype-to-phenotype transformations [reviewed by Ghosh et al. (2011); Lucas et al. (2011); Papp et al. (2011)]. We expect that this systematic exploration of the systems biology of domestication will lead to an enriched, mechanistic view of both the nature of “adaptation” and of “parallel evolution.”

### Conflict of interest statement

The authors declare that the research was conducted in the absence of any commercial or financial relationships that could be construed as a potential conflict of interest.
